# Colostrum from Cows Immunized with a Vaccine Associated with *Bovine Neonatal Pancytopenia* Contains Allo-Antibodies that Cross-React with Human MHC-I Molecules

**DOI:** 10.1371/journal.pone.0109239

**Published:** 2014-10-09

**Authors:** Rahel Kasonta, Mark Holsteg, Karin Duchow, James W. Dekker, Klaus Cussler, Justin G. Bendall, Max Bastian

**Affiliations:** 1 Division of Veterinary Medicine, Paul-Ehrlich-Institut, Langen, Germany; 2 Fonterra Research and Development Centre, Palmerston North, New Zealand; 3 Landwirtschaftskammer Nordrhein-Westfalen, Referat 34 Tiergesundheit, Bonn, Germany; Emory University School of Medicine, United States of America

## Abstract

In 2006, a new haemorrhagic syndrome affecting newborn calves, *Bovine Neonatal Pancytopenia* (BNP), was reported in southern Germany. It is characterized by severe bleeding, destruction of the red bone marrow, and a high case fatality rate. The syndrome is caused by alloreactive, maternal antibodies that are ingested by the calf with colostrum and result from a dam vaccination with one particular vaccine against Bovine-Viral-Diarrhoea-Virus. Because bovine colostrum is increasingly gaining interest as a dietary supplement for human consumption, the current study was initiated to elucidate whether BNP alloantibodies from BNP dams (i.e. animals that gave birth to a BNP-affected calf) cross-react with human cells, which could pose a health hazard for human consumers of colostral products. The present study clearly demonstrates that BNP alloantibodies cross-react with human lymphocytes *in vitro*. In agreement with previous reports on BNP, the cross-reactive antibodies are specific for MHC-I molecules, and sensitize opsonised human cells for *in vitro* complement lysis. Cross-reactive antibodies are present in serum and colostrum of individual BNP dams. They can be traced in commercial colostrum powder manufactured from cows immunized with the vaccine associated with BNP, but are absent from commercial powder manufactured from colostrum excluding such vaccinated cows. In humans alloreactive, MHC-I specific antibodies are generally not believed to cause severe symptoms. However, to minimize any theoretical risk for human consumers, manufacturers of bovine colostrum for human consumption should consider using only colostrum from animals that have not been exposed to the vaccine associated with BNP.

## Introduction

During the past two decades, bovine colostrum has gained increasing interest as a dietary supplement for human consumption. Several studies have proposed beneficial effects for colostral, antimicrobial antibodies [1]–[3], while other studies have postulated that small molecules such as peptidic growth factors may have an advantageous influence on gastrointestinal disorders [4]. Consequently, international food and pharmaceutical companies have developed an array of different colostrum based products ranging from sports food [5] to dietary supplements aiming to ameliorate unspecified diarrhea in AIDS patients [6]. Annual sales of dry colostrum ingredients reached a volume of 2,600 tonnes with a value of US$80 million in 2007 [7].

However, in 2006 a new hemorrhagic syndrome of bovine neonates, *Bovine Neonatal Pancytopenia* (BNP) was first observed in Central European countries. The syndrome affects newborn calves and is characterized by a complete destruction of the red bone marrow, pancytopenia, severe bleeding and high lethality. The syndrome is triggered by ingestion of colostrum from dams that have previously been vaccinated with PregSureBVD, a strongly adjuvanted, inactivated vaccine against bovine viral diarrhea virus (BVDV) [8], [9]. Due to its particular composition that combines substantial amounts of bioprocess impurities with a very efficient adjuvant system, this vaccine induces high titres of bovine MHC-I-specific alloantibodies [10]–[12]. Transfer of alloreactive, maternal antibodies via colostrum to a newborn calf carrying the corresponding alloantigens leads to severe pancytopenia [13]. The pathoetiology of BNP is therefore similar to human alloimmune diseases such as the Rhesus-Incompatibility Syndrome or Neonatal Alloimmune Thrombocytopenia (NAIT), except that such diseases involve transfer of alloimmune antibodies to the fetus *in utero* rather than via breast milk to neonates.

Due to the compelling evidence for the causal role of PregSure BVD vaccine in the induction of BNP, the European Medicines Agency (EMA) suspended the marketing authorisation for PregSure in 2010 [14]. Shortly after the problem had been identified in Europe, a number of BNP cases were observed in New Zealand leading to a withdrawal of the vaccine from the New Zealand market [15].

This animal disease, amongst calves, raises questions about the safety of such colostral products for human consumption. Therefore a joint study was initiated between the Fonterra Research and Development Centre and the Paul-Ehrlich-Institute (PEI) to clarify whether the New Zealand haemorrhagic cases amongst calves were due to BNP and to assess whether the colostrum of the respective dams contained antibodies capable of cross-reacting with human cells.

## Materials and Methods

### 1. Serum and dairy samples

Two sets of serum samples from 8 and 4 PregSureBVD-vaccinated New Zealand BNP dams were obtained. During the calving season 2011 these cows gave birth to calves, which developed BNP within the first three weeks of life. Sera from PregSureBVD vaccinated animals with no history of BNP in their progeny (n = 79) and from non-vaccinated (n = 20) or alternatively BVDV-vaccinated animals (n = 20) served as controls. In the herds with BNP-affected calves, the vaccination programme consisted of two doses given 4–6 weeks apart, followed by annual administration of a booster dose. The animals sampled had received a minimum of three vaccinations of PregSureBVD in the years preceding the production of a BNP-affected calf. The majority of cows was of Holstein-Frisian breed. In New Zealand some cows were Holstein-Frisian Jersey crossbreeds. The collection of blood samples from NZ dairy cows was being undertaken by veterinarians as part of routine disease investigations. In addition, milk and colostrum samples were obtained from 12 and six, respectively, of the BNP dams. Individual colostrum samples investigated in this study were obtained from one of the first four milkings after parturition. Raw milk and colostrum samples from individual cows were subjected to continuous-flow High Temperature Short Time pasteurization by: using a peristaltic pump to pass small volumes (600 ml in total) through a miniature plate heat exchanger (PHE), fitted to a hot-water heating supply; then through a holding tube with the peristaltic pump adjusted such that the flow rate provided a residence time of 15 seconds; then past an electronic temperature probe to ensure that the flow of hot water to the initial PHE was adjusted such that the temperature of the liquid within the far end of the holding tube was at least 72.6°C; and finally through a second miniature PHE, fitted to a iced-water cooling supply; and then discarding the first 250 mL portion that emerged, prior to collecting each sample. Commercial lots of New Zealand whole milk powder and two colostrum powders were also obtained. For the production, milk and colostrum were pasteurized and then spray dried to powder. The colostrum powder was manufactured from the factory located in the region of New Zealand, which had the greatest proportion of dairy herds using PregSureBVD vaccination. The colostrum used to manufacture colostrum powder was pooled from across all cows in a herd into each farm vat, and then pooled from multiple herds' farms vats. So finished colostrum powder used in this study represents a colostrum blend from several thousand cows. Although the first eight milkings after parturition may consist of colostrum, for the production of commercial colostrum powder only colostrum collected from the first four milkings was used. One colostrum powder batch was manufactured in New Zealand in 2011, prior to the prohibition against PregSureBVD treatment, while the second sample was manufactured in New Zealand in 2012 from colostrum that had been sourced exclusively from non-PregSure-treated cows. All samples were held at -70°C for long term storage. The powders were weighed and 0.1 g each was dissolved in 1 ml phosphate buffered saline (PBS, PEI), prepared fresh before each analysis. Whole colostrum was centrifuged twice at 11,000×g followed by 25,000×g to remove cell debris, and stored at −20°C. For comparison, serum samples of six European BNP dams form North-Rhine-Westphalia were included in the study. According to the new German animal welfare legislation, the study was announced to and approved by the competent authority (State Office for Nature, Environment and Consumer Protection of North-Rhine-Westfalia, LANUV Recklinghausen, Germany; ref. 84-02.05.40.14.032).

### 2. Cell preparation and cell culture

Bovine leukocytes were prepared from whole blood of healthy heifers by ammonium chloride lysis and Ficoll gradient centrifugation as described elsewhere [10]. Short-term T cell lines were obtained from peripheral blood mononuclear cells (PBMCs) by phytohaemagglutinin (PHA) stimulation as previously described [10]. The resulting polyclonal T cell lines are hereafter referred to as lymphoblasts. Human lymphoblasts were prepared accordingly from buffy coats of healthy blood donors. The buffy coats were purchased from the German Red Cross Bloodbank, Frankfurt. All donors have given informed consent to the medicinal, scientific or pharmaceutical use of their blood or preparations thereof. Before distribution the material is anonymized. To the customer no information is disclosed about individual donors. This procedure has been approved by the local ethics committee (Votum 329/10; ethics committee; Goethe-University, Frankfurt).

The bovine kidney cell line used for the production of PregSureBVD, hereafter referred to as BK cell line, was kindly provided by Pfizer Animal Health [10]. The cell line was tested to be free of BVDV and was maintained according to manufacturer's instructions as previously described [10].

### 3. Flow cytometry

**Figure 1:**
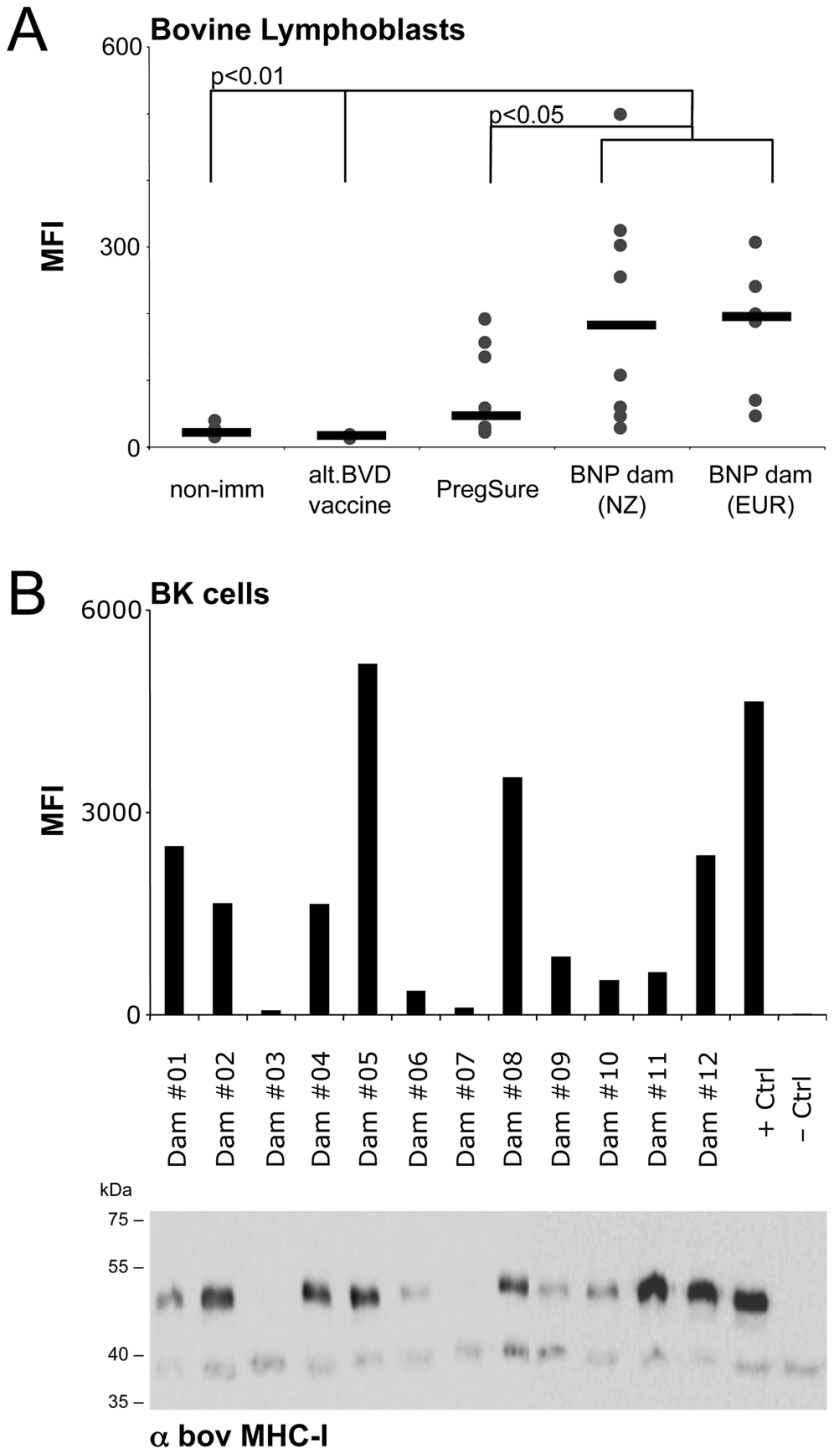
Sera of NZ-BNP dams show identical alloreactivity compared to European BNP dams. (A) Sera were obtained from NZ dairy cows that had either not been immunized against BVDV (non-BVD-imm.), vaccinated with an alternative BVD vaccine (BVD-vaccine), vaccinated with PregSureBVD and birthed healthy calves (PregSure) or cows that had been vaccinated with PregSureBVD and gave birth to BNP calves (BNP dams (NZ)). By flow cytometry a first set of eight sera per group was tested for alloreactive binding to bovine lymphoblasts. As a control we included six serum samples of European BNP dams (BNP dam (EUR)). Symbols represent the median fluorescence intensity (MFI) for individual serum samples, black bars indicate the median value for each group. (B) Using BK cells, *i.e*. the cell line used for the production of PregSureBVD, the full panel of serum samples from twelve NZ BNP dams were tested in parallel by flow cytometry and immunoprecipitation for the presence of BNP associated alloantibodies. The upper panel shows the MFI for the individual serum samples, the lower panel shows the corresponding immunoprecipitate as revealed by a monoclonal antibody specific for bovine MHC-I molecules. Serum from a European BNP dam served as a positive control, fetal calf serum was used as a negative control.

**Figure 2:**
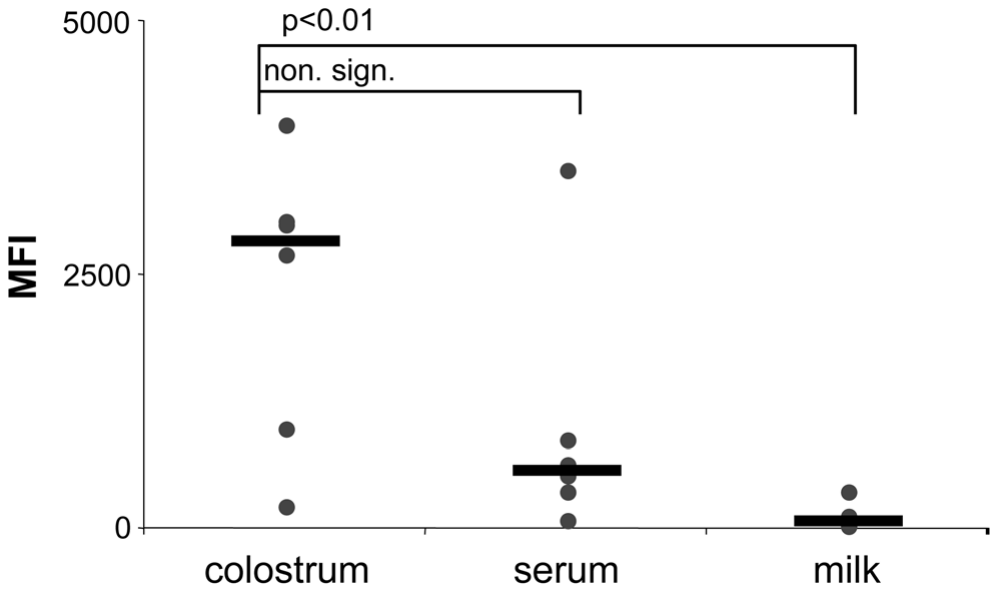
Comparison of BNP-associated alloantibody content in BNP dam colostrum, serum and milk. Colostrum, serum and milk samples were collected from six NZ BNP dams. The samples were diluted 1:500. Using BK cells the samples were tested by flow cytometry for the presence of BNP associated alloantibodies. Symbols represent the median fluorescence intensity (MFI) for individual samples as determined by flow cytometry, black bars indicate the median value for each group.

**Figure 3:**
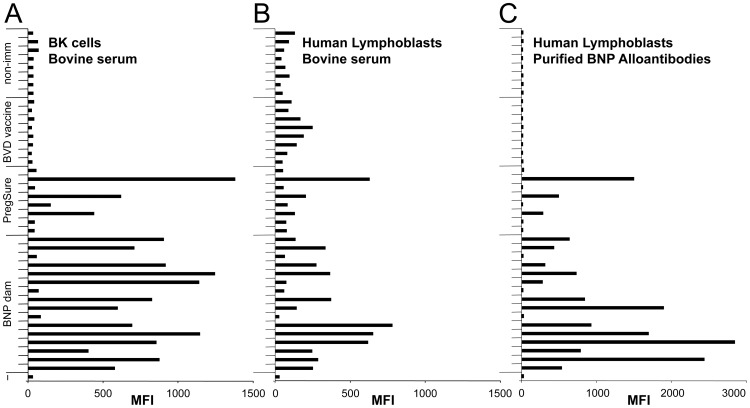
PregSureBVD induced BNP alloantibodies cross-react with human lymphoblasts. (A) A panel of sixteen BNP dam sera was tested by flow-cytometry for the presence of alloreactive antibodies that bind to the BK cell line. As controls sera from non-immunized, alternatively vaccinated or PregSureBVD-immunized non-BNP dams were included. (B) The same serum panel was tested for cross-reactive binding to human lymphoblasts. (C) From the entire serum panel BNP-associated alloantibodies were purified by affinity purification. The affinity purified alloantibodies were again tested for cross-reactive binding to human lymphoblasts. Black bars indicate the MFI obtained with individual sera or the corresponding affinity-purified alloantibodies.

**Figure 4:**
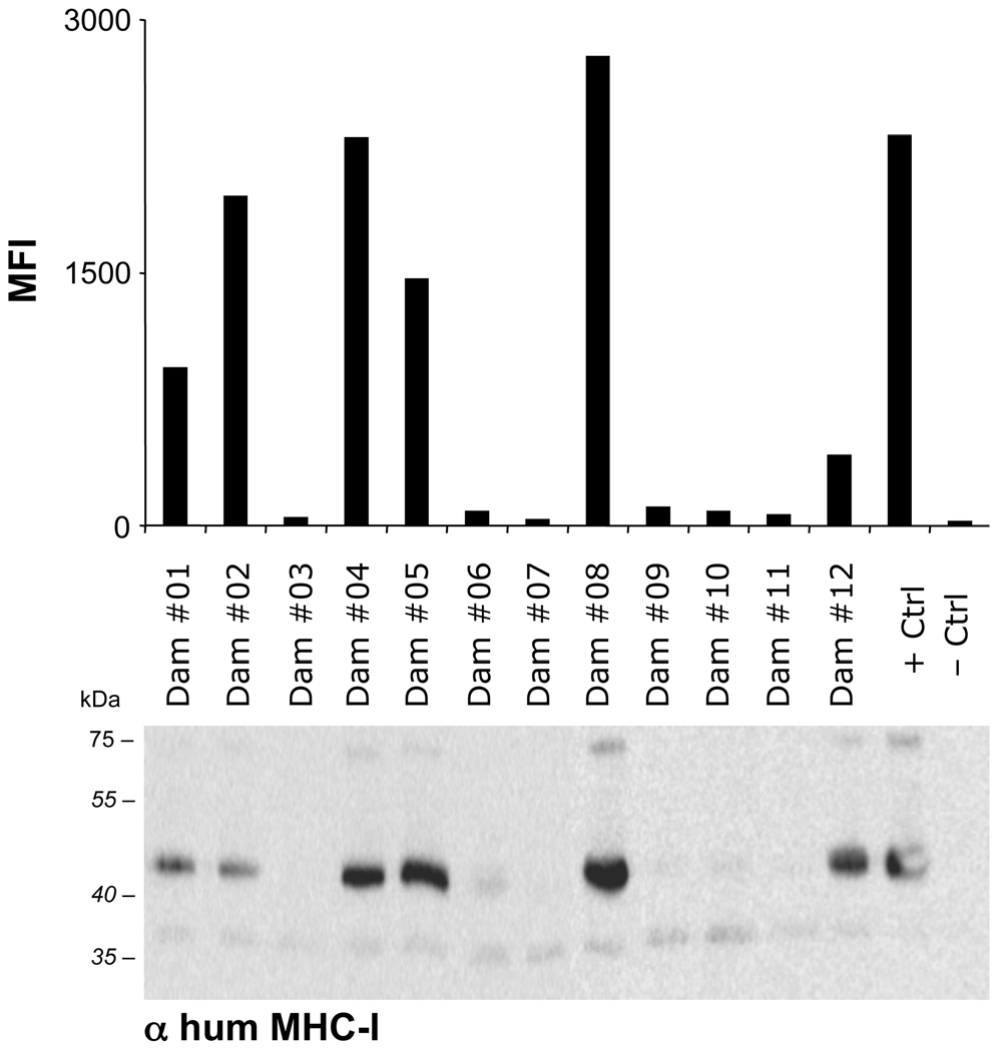
BNP-associated alloantibodies bind human MHC-I molecules. The panel of BNP dam sera was tested in parallel by flow-cytometry and by immunoprecipitation using human lymphoblasts. The black bars in the upper panel show the MFI as determined by flow cytometric analysis. The lower panel shows the corresponding immunoprecipitates as revealed by a monoclonal antibody specific for human MHC-I molecules.

**Figure 5:**
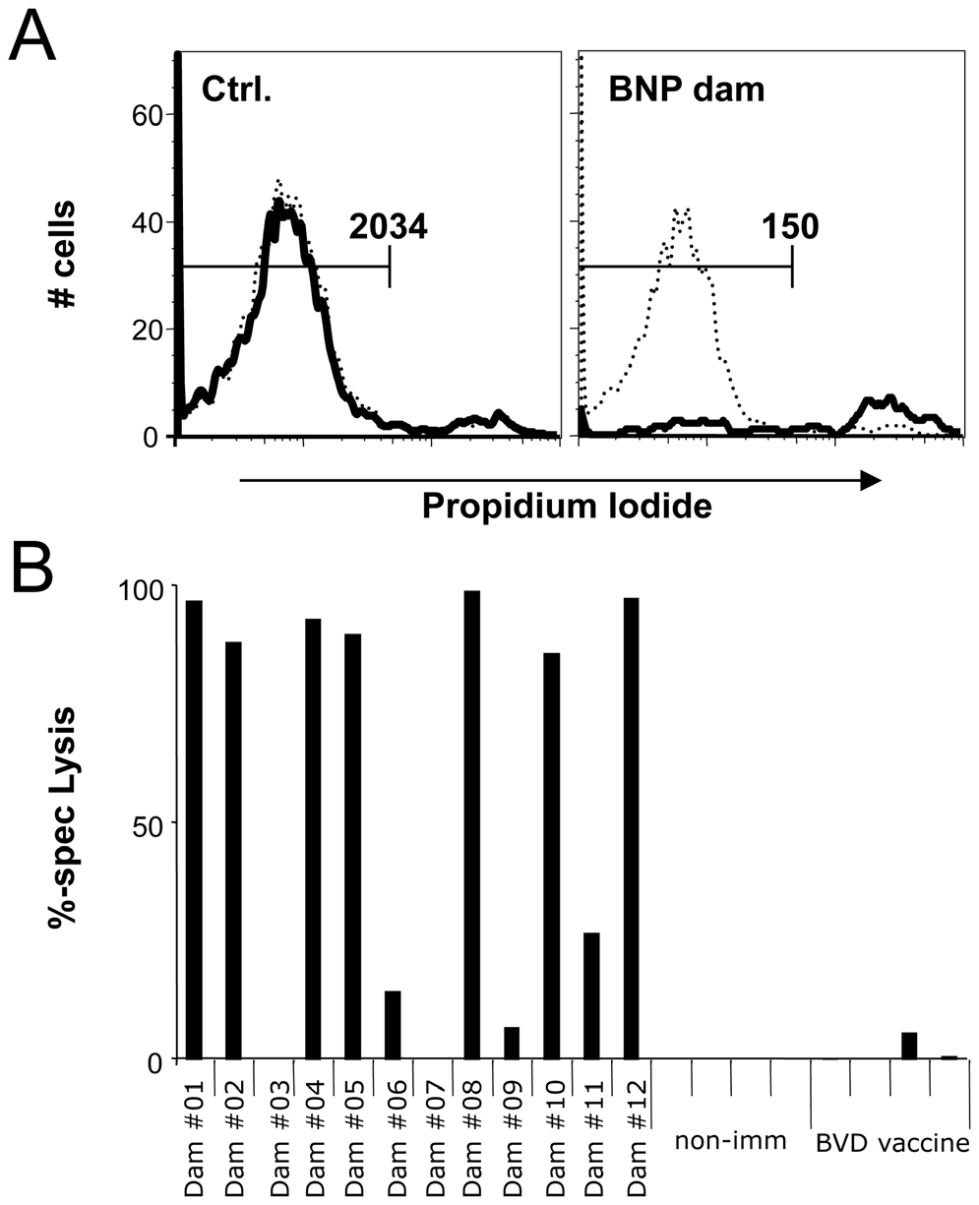
BNP-associated alloantibodies opsonise and sensitize human lymphoblasts for complement-mediated cell lysis. Human lymphoblasts were coincubated with heat-inactivated bovine serum and rabbit complement was added. Cell viability was measured by flow cytometry counting live, propidium-iodide negative cells. (A) The histograms show human lymphoblasts incubated with serum from a non-PregSureBVD immunized control dam (left) or BNP dam serum (right) after adding active complement (bold line). As a control, heat inactivated rabbit complement was added (dotted line). Numerical figures indicate the absolute number of living, propidium-iodide negative cells. (B) The serum panel was tested for its complement sensitizing activity on human lymphoblasts. Sera from four non-immunized and four alternatively vaccinated animals served as a control. One representative of three experiments is shown. Black bars represent the specific lysis for the individual sera.

**Figure 6:**
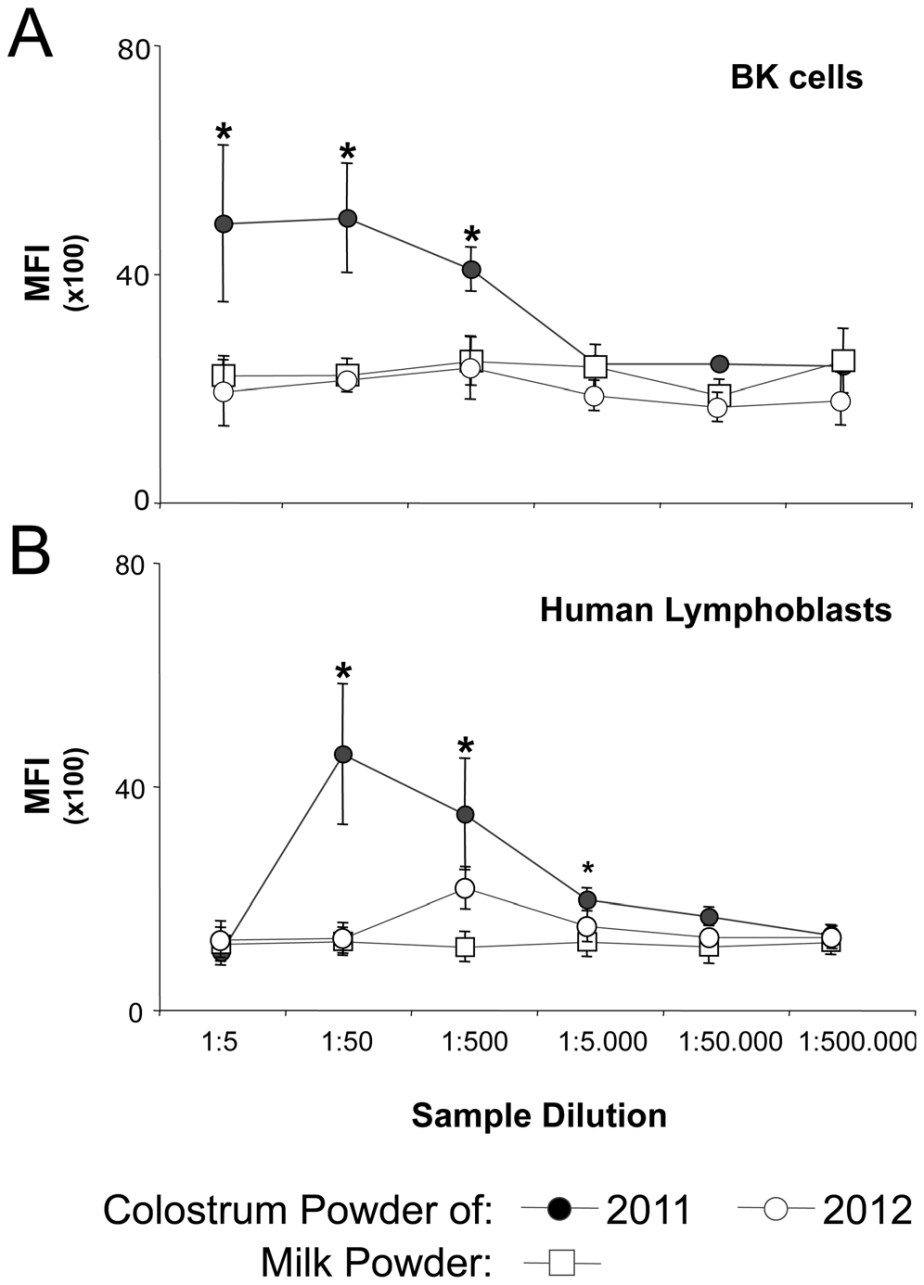
Exclusion of PregSureBVD immunized animals reduces the amount of cross-reactive antibodies in commercial colostrum powder. Two commercial lots of colostrum powder were compared by flow cytometry to whole milk powder for the presence of BNP-associated alloantibodies, one lot was produced during the calving season of 2011, the other was produced in 2012 according to a new harvesting policy excluding herds that had been vaccinated with PregSurerBVD. Filled circles represent the MFI for colostrum powder produced in 2011, open circles for 2012 colostrum, open quadrates for whole milk powder. Panel (A) depicts the reactivity for BK cells, panel (B) the reactivity to human lymphoblasts. Symbols represent the median over three independent flow cytometric analyses, error bars the corresponding standard deviation. Asterisks indicate a significant difference between the two colostrum batches at the indicated dilution. For the analyses an Accuri C6 flow cytometer was used.

To examine samples for the presence of opsonising alloimmune antibodies, flow cytometry analyses were carried out as previously described [10]. Briefly, up to 1×10^5^ BK cells or lymphoblasts were re-suspended in PBS containing 0.5% fetal calf serum (FCS, Gibco). Sera, colostrum or milk and also individually pasteurized colostrum samples and reconstituted commercial milk and colostrum powders were added to a final dilution of 1∶5 (if not stated otherwise) followed by one hour incubation at 4°C. Cells were then washed twice with PBS containing 0.5% FCS and cell surface bound bovine IgG detected using a FITC-conjugated polyclonal sheep-anti-bovine IgG antibody (Invitrogen). Median fluorescence intensity (MFI) of living cells as defined by Forward-Scatter (FSC)/Sideward-Scatter (SSC)-gating was determined for each sample using a BD Lsr II Flow Cytometer (Figs 1–4) or a BD Accuri C6 Flow Cytometer (Figs 5–6). The Accuri device uses a different log scale to display fluorescence data, which results in a baseline shift of two log scales between the two devices. To take this into account, figures using Accuri data were given an adapted scale (as indicated in the axis title, which now reads: MFI (×100)).

### 4. Immunoprecipitation

To isolate and identify BNP associated alloantigens on BK cells or lymphoblasts immunoprecipitation (IP) was performed as previously described [16]. In summary, cells were surface labeled with EZ-Link Sulfo-NHS-LC-Biotin (Thermo Scientific) and incubated with bovine serum samples. Cells were then solubilized with Lysis Buffer (1% Triton X-100, 150 mM NaCl, 50 mM Tris and 5 mM EDTA) in an ultrasonic bath. Solutions were clarified by centrifugation (10,000×g, 10 min) and the immune complexes precipitated with protein G-sepharose beads (GE Healthcare), eluted with Loading Buffer containing 6 M urea, 62.5 mM Tris, 2% SDS, 10% glycerol and 0.025% bromophenol blue, and subjected to SDS-PAGE under non-reducing conditions.

Proteins were blotted onto nitrocellulose membranes (Whatman). Biotinylated cell membrane proteins were detected using a Streptavidin-HRP conjugate (Dianova) and visualized using enhanced chemoluminescence (ECL; GE Healthcare) reagents. MHC-I molecules were visualized by developing the membranes with an anti-bovine MHC I antibody (IL-A88; AbD Serotec) or an anti-HLA I antibody (w6/32, kindly provided by Steffen Tenzer, University of Mainz) followed by a secondary anti-murine-IgG^HRP^ conjugate (Dianova).

### 5. Affinity purification of BNP alloantibodies

BNP alloreactive antibodies were affinity purified as previously described [10]. Sera of PregSureBVD vaccinated dams, alternatively vaccinated animals, or non-immunized controls were diluted 1∶10 in PBS and incubated with BK cells. The cells were repeatedly washed with 0.9% sodium chloride. Cell surface-bound antibodies were detached by treatment with ice-cold citrate buffer (0.12 M sodium citrate, pH 2.5) for 15 minutes. After centrifugation (10,000×g, 5 min) supernatants were harvested and immediately treated with neutralization buffer (1 M Tris HCl, pH 9.0).

### 6. Flow cytometric detection of complement-activating antibodies

To assess the complement activating activity of opsonising alloantibodies, we adopted a flow cytometry based, complement-dependent cytotoxicity assay [17]. Briefly, 1×10^5^ human lymphoblasts, re-suspended in PBS containing 0.5% FCS, were incubated with heat inactivated (56°C, 30 minutes) bovine sera at a final dilution of 1∶5 and incubated for 45 minutes at 4°C. Complement activation was assessed by adding active or heat-inactivated rabbit complement, *i.e*. fresh rabbit serum. After 10 minutes at 37°C, propidium iodide (PI, Sigma-Aldrich) was added, the samples were placed on ice and immediately analysed by flow cytometry. The cytometer settings were limited to run a fixed volume of 20 µl. For each serum sample the number of living, PI-negative cells (N) after incubation with active or heat-inactivated rabbit complement was determined. The percentage of cytotoxicity was calculated according to the following equation: specific Lysis  = 100% x (1 – N_active complement_/N_inactive complement_).

### 7. Statistical analysis

Single-tailed, paired or unpaired, homoskedastic Student's t test was performed to test for significant differences between groups, indicated by the p-value in the respective graphs (significance level α = 0.05). Pearson's test was used to calculate correlation coefficients.

## Results

### 1. New Zealand dams with BNP calves have high alloreactive antibody titres

During the 2011 calving season, several New Zealand farms experienced haemorrhagic disorders in bovine neonates. To determine whether BNP, the vaccine-induced alloimmune syndrome that had previously been described only in Central Europe, was the underlying cause, serum samples from affected dairy herds and unaffected controls were collected and investigated for the presence of alloreactive antibodies by flow cytometric assay. Bovine lymphoblasts obtained from an adult Holstein-Frisian cow were incubated with respective sera and cell surface-bound alloantibodies quantified by flow cytometry. Living cells were identified according to their FSC/SSC characteristics and median fluorescent intensity (MFI) was determined for the living cell population (Fig S1). The MFI served as a correlate for the amount of alloantibodies present in each serum sample. MFI obtained from New Zealand BNP dam sera was similar to the confirmed BNP cases from German dams (Fig 1A). In accordance with previous observations made in Central Europe [10], we found that BNP dams had significantly higher levels of alloantibodies than PregSureBVD vaccinated animals giving birth to calves that did not develop BNP, or animals that received an alternative BVDV vaccine, or non-vaccinated controls (Fig 1A).

To confirm that bovine MHC-I molecules were targeted by the vaccine induced alloantibodies, as previously described [11], [12], [16], BNP dam sera were tested by immune precipitation. BK cells (i.e. the cell line used in the production of PregSureBVD) were biotinylated and incubated with each test sera. Cell surface molecules complexed to the alloantibodies were precipitated, separated by SDS-PAGE, blotted and visualized by peroxidase-conjugated streptavidin (data not shown) or MHC-I-specific staining. In parallel, the same samples were tested against BK cells by flow cytometry. The level of alloantibody binding as measured by flow cytometry correlates significantly with the amount of bovine MHC-I detected, as revealed by the presence of a Western blot band of approximately 43 kDa (Fig 1B and Fig S2A and S3). However, in two out of the 12 BNP dam sera, no alloantibody binding was detectable. Whether this is due to serum degradation, or whether these calves suffered from BNP-like symptoms not related to PregSureBVD induced MHC-I alloantibodies, is not clear.

### 2. BNP associated alloantibodies are concentrated in colostrum

Bovine colostrum represents an antibody concentrate that supplies the neonate with maternal antibodies, and it has been shown that BNP-associated alloantibodies are present in the colostrum of BNP dams [13]. To assess the amount of BNP-associated alloantibodies in colostrum and milk, matched samples of: colostrum, serum and milk from six individual New Zealand BNP dams were tested. We found that the reactivity to BK cell surface molecules was highest in colostrum, followed by serum, while only low to negligible amounts of BNP-associated alloantibodies were present in milk. Presumably, due to one outlier the difference between colostrum and serum is not significant (Fig 2). To test whether pasteurization potentially reduces the activity of colostrum contained alloantibodies, individually pasteurized colostrum samples were compared to unpasteurized colostrum samples. However, no significant change in binding capacity of the alloantibodies could be detected (data not shown). Colostrum is only produced during the first days after birth, whereas the first clinical signs of BNP occur ten days after birth making it challenging to obtain colostrum from BNP dams. Since the alloreactivity was sufficiently detectable in both colostrum and serum, we performed the majority of the subsequent experiments on serum samples.

### 3. BNP-associated alloantibodies cross-react with human lymphoblasts

To investigate whether BNP-associated alloantibodies cross-react with human cells, we compared the surface-binding reactivity of our serum panel to the BK cell line with human lymphoblasts. There was a significant correlation (R = 0.71) of the reactivity to BK cells and to human lymphoblasts (Fig 3A and B). To confirm that this reactivity was due to PregSureBVD vaccination, the experiment was repeated using affinity-purified, vaccine-induced alloantibodies. While affinity-purified alloantibodies from PregSureBVD-vaccinated animals, i.e. three out of eight PregSureBVD immunized non-BNP and thirteen out of sixteen BNP dams, exhibited the same cross-reactivity pattern to human lymphoblasts as the sera, the minor reactivity seen with sera from animals not vaccinated with PregSureBVD disappeared indicating that those signals were non-specific. To identify the antigen BNP-associated alloantibodies recognized on human lymphoblasts, we performed immunoprecipitation experiments using human lymphoblasts. Again, for BNP dam sera the flow cytometric reactivity correlated significantly with the precipitation of human MHC I (Fig 4 and Fig S2B), while sera from non-immunized or alternatively vaccinated animals displayed no MHC I-specific reactivity (data not shown).

It has been speculated that complement-mediated lysis of alloantibody-opsonised cells contributes to the pathogenesis of BNP [10], [18]. To assess whether BNP-associated alloantibodies could potentially destroy human lymphocytes through such a mechanism, we adapted a flow-cytometric approach to measure complement activity *in vitro* [17]. All ten BNP sera that contained alloreactive antibodies induced a specific complement-lysis, while the corresponding control sera showed no effect (Fig 5). This indicates that BNP-associated alloantibodies not only recognize human MHC-I molecules, but also have the capacity to exert a cytotoxic effect on human lymphoblasts. The same observation was made on BK cells with PregSureBVD induced, MHC-I reactive alloantibodies from non-BNP and BNP dams (Fig S3 and S4).

### 4. BNP Alloantibodies are present in commercially available dairy products

To test whether BNP-associated alloantibodies are present in commercial lots of colostrum powder, the material used by down-stream industries to produce colostrum based dietary supplements for human consumption, we investigated milk and colostrum powders that had been produced during and after the 2011 calving season. Flow cytometry showed significant reactivity against BK cells and human lymphoblasts in the 2011 colostrum powder lot (Fig 6), although due to a production-inherent dilution effect the binding is much lower compared to the reactivity of individual BNP dam colostra (see Fig 2 for comparison). In view of this observation, Fonterra withheld all 2011 colostrum from sale, and in 2012 changed the colostrum harvesting policy to exclude all animals that had been vaccinated with PregSureBVD. Colostrum powder manufactured during the 2012 calving season displayed no cross-reactivity to BK cells or human lymphoblasts.

## Discussion

The aim of the current study was to clarify whether cases of bovine neonate haemorrhages in New Zealand were caused by the vaccine induced feto-maternal incompatibility syndrome, BNP. Furthermore, the question was addressed whether the haemorrhage causing alloantibodies in the colostrum of PregSureBVD immunized cows could cross-react with human cells and might therefore pose a theoretical risk to human consumers of products manufactured from such colostrum.

The data presented in the current study are consistent with the contention that the cases of bovine neonate haemorrhage observed in NZ were caused by BNP. The alloreactivity in sera of NZ dairy cows showed the same reactivity pattern as that previously observed in Europe [10], [13]. Only sera from PregSureBVD immunized animals showed alloreactive binding to bovine lymphocytes, and this alloreactivity was directed against MHC-I molecules of BK cells. Although this alloreactivity was present in the serum, it was clearly higher in the colostrum of BNP dams. This is in concordance with observations in Europe and was expected because, in ruminants, colostrum is the only source of maternal antibodies and contains high amounts of immunoglobulins [3]. Compared to serum or colostrum, the level of alloantibodies in milk was negligible. From this observation there is no evidence that the consumption of milk from BNP dams is hazardous for human consumers.

By contrast, for colostrum or serum our data clearly prove that BNP-associated alloantibodies cross-react with human MHC-I molecules. Whether this could potentially be hazardous for human consumers, cannot be conclusively answered in this study: In humans, alloimmune syndromes with severe clinical signs are generally attributed to specific alloantigens that are expressed on particular target cell types only. For example, NAIT is induced in most cases by alloantibodies targeting a dimorphic epitope in the beta chain of Human Platelet Antigen 1 (HPA-1a), which is only present on megakaryocytes and thrombocytes [19]. The particular expression pattern of the alloantigen explains the specific insult of NAIT antibodies on the haemostatic system [20]. Although MHC-I specific antibodies have originally been implicated in the induction of NAIT, it was later shown that they rather play an inferior role [20]. In the case of BNP no specific target antigen other than MHC-I has yet been identified. Instead, there is evidence for a causal role of MHC-I specific alloantibodies. Several studies have identified MHC-I specificity as the only common denominator in the serum of BNP dams [11], [12], [16], [21]. Furthermore, Bridger *et al*. demonstrated in an elegant study that the surface opsonising activity of orally administered BNP dam antibodies — which has been shown to correlate with MHC-I reactivity — is directly associated with the severity of clinical symptoms in challenged calves [13]. Finally, we observed in two cases that fraternal twins of BNP-affected calves, expressing MHC-I molecules that were not recognized by the alloantibodies of the dam, survived the ingestion of toxic colostrum and exhibited a completely normal appearance of their red bone marrow [22]. So, one important current hypothesis is that MHC-I specific alloantibodies cause BNP in bovines.

However, despite the complement activating effect on human lymphoblasts observed *in vitro*, it is questionable whether the bovine alloantibodies could cause any effect in humans. The main difficulty with a pathoetiological role of MHC-I-specific antibodies is that MHC-I proteins are ubiquitously expressed on the vast majority of cells. Even though the actual pathomechanism leading to cell destruction after alloantibody intake is not elucidated, it can be assumed that large amounts of MHC-I alloantibodies would be required to saturate peripheral MHC-I before such antibodies could reach a tissue and induce a specific insult. It is conceivable that with the sudden uptake of several grams of maternal antibodies in calves, such levels of alloantibodies are reached. However, it is improbable for such a massive influx to occur in humans. Firstly, in contrast to bovine neonates, bovine dietary antibodies are not effectively transported across the human gut mucosa. The neonatal uptake of maternal antibodies via the gut mucosa is mediated by the neonatal Fc-receptor, FcR_n_, a dedicated transport system that shuttles maternal antibodies across the gut-blood barrier. It is known that the mucosal barrier closes 24–48 hrs after birth and at least in rodents it has been shown that FcR_n_ is downregulated directly after birth [23]. In humans, the fetus is provided with maternal antibodies via the placenta during gestation [24], [25]. While the neonatal Fc-receptor is expressed mainly in the fetal placenta, it is also found in the gut mucosa [26] where it is expressed throughout life [27], [28]. *In vitro* studies have shown that whilst the expressed receptor is functional and transports antibodies across polarized cell layers [29], the main function of human FcR_n_-expression in the gut is to shuttle antibodies from the submucosal side into the gut lumen [30]. The direction of the net transport is determined by the IgG concentration gradient [26]. Since the affinity of the human FcR_n_ for human IgG is four-fold higher than that of bovine IgG [31], and since the concentration of cross-reactive alloantibodies in commercial colostrum products is relatively low, the uptake of cross-reactive alloantibodies is inefficient, such that only insignificant amounts of MHC-I-specific BNP antibodies would reach the circulation.

Secondly, there are cases where human breast milk can contain alloreactive antibodies. For instance, for NAIT, platelet-specific antibodies have been identified in the breast milk of affected mothers, but this breast milk is still considered safe to consume by the infant [32]. Similarly for other maternal autoimmune diseases, such as lupus or Hashimoto's thyroiditis, disease manifestation in the infant is due to maternal alloantibodies transferred *in utero* rather than via lactation, such that disease exacerbation is not associated with breast feeding [33], [34]. This suggests that alloantibodies present in the infant diet are unlikely to result in detrimental health effects.

Taking all these observations and considerations together, we conclude that the risk of colostrum-containing dietary supplements, from cows treated with the vaccine associated with BNP, is low for human consumers. However, since the reactivity of BNP alloantibodies to human lymphoblasts is principally indistinguishable from the reactivity to bovine cells, a residual risk cannot be absolutely excluded. In what may have been an overly cautious approach, Fonterra decided to withhold the entire colostrum production of 2011, and from 2012 onwards excluded colostrum collection from PregSure-treated cows. This approach was effective, as no alloreactivity to bovine or human cells was detectable in colostrum powder manufactured according to the new supply policy.

## Supporting Information

Figure S1 **The gating strategy of the flow-cytometric analysis.** BK cells (A) and bovine (B) or human lymphoblasts (C) were incubated with serum from a non.-immunized cow (punctuate line) or a BNP dam (bold line). Cells were washed twice and incubated with a FITC-conjugated secondary antibody. Subsequently, cells were analyzed by flow-cytometry. Live cells were identified and gated according to their FSC/SSC characteristics (black lined area in the left panels). The median fluorescence intensity was analyzed for all events within the live cell gate (right panels).
(PDF)

Figure S2 **Alloantibody reactivity measured by FACS correlates with MHC-I immunoprecipitation.** The intensity of immunoprecipitated MHC-I bands shown in Fig 1B and Fig 4 was analyzed by densitometry. The arbitrary grey value of the densitometry was plotted against the corresponding alloantibody binding as determined by flow-cytometry (MFI). The correlation is shown for BK cells (A) and for human lymphoblasts (B). Pearson correlation coefficients and p-values are indicated.
(PDF)

Figure S3 **Alloantibody reactivity measured by FACS correlates with MHC-I immunoprecipitation.** (A) BK cells were incubated with eight sera of non-immunized or alternatively BVD-vaccinated cows or of PregSureBVD-vaccinated non-BNP dams and PregSureBVD vaccinated BNP dams. Surface molecules were immunoprecipitated as described. The precipitates were analyzed by SDS-PAGE followed by anti BoLA-I westernblot using monoclonal antibody IL A88. Specific MHC-I bands at about 40 kDa (lined area) were analyzed by densitometry. (B) The same panel of sera was tested in parallel by flow-cytometry for alloantibody binding. MFI values (black bars) are plotted side by side with the corresponding grey values as determined by densitometry (grey bars). The correlation was calculated, the respective Pearson coefficient and the p-value are indicated.
(PDF)

Figure S4 **Alloantibody mediated complement lysis correlates with FACS reactivity.** (A) BK cells were incubated with serum from a non-immunized cow (left panel) or a BNP dam (right panel). Heat-inactivated (dotted line) or active rabbit complement (bold line) was added and samples were incubated at 37°C. To identify dead cells red fluorescent propidium iodide was added and samples were analyzed by FACS. The absolute number of living, propidium iodide negative cells in a defined sample volume of 20 µl was determined. Numerical figures in the graphs represent the respective number of living cells after adding active complement. (B) Specific cell lysis was determined for the same serum panel as in Fig S3 and tested in parallel by flow-cytometry for alloantibody binding. MFI values (black bars) are plotted side by side with the corresponding %-specific cell lysis (open bars). The correlation was calculated, the respective Pearson coefficient and the p-value are indicated.
(PDF)
