# Supplementary figures and images for: Colostrum from Cows Immunized with a Vaccine Associated with Bovine Neonatal Pancytopenia Contains Allo-Antibodies that Cross-React with Human MHC-I Molecules
Source: PLoS One. 2014 Oct 9;9(10):e109239. doi: 10.1371/journal.pone.0109239 (PMC4192356; doi:10.1371/journal.pone.0109239)

Figure S1

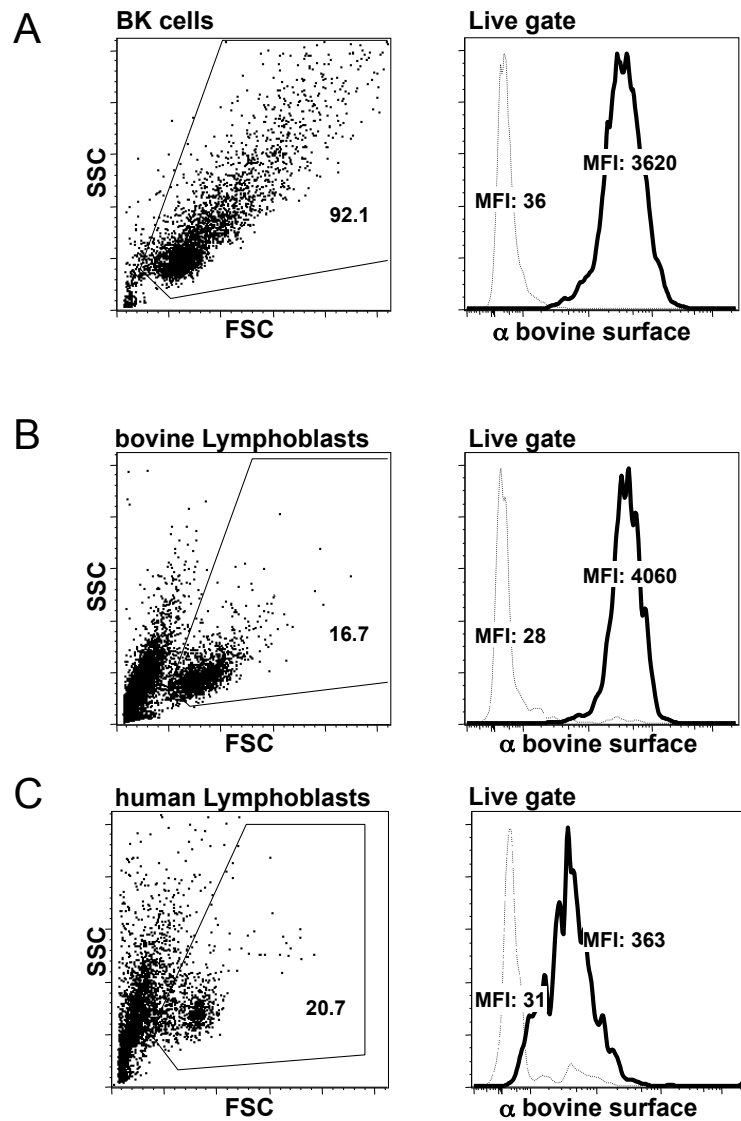

Supplement: Figure S1 — The gating strategy of the flow-cytometric analysis. BK cells (A) and bovine (B) or human lymphoblasts (C) were incubated with serum from a non.-immunized cow (punctuate line) or a BNP dam (bold line). Cells were washed twice and incubated with a FITC-conjugated secondary antibody. Subsequently, cells were analyzed by flow-cytometry. Live cells were identified and gated according to their FSC/SSC characteristics (black lined area in the left panels). The median fluorescence intensity was analyzed for all events within the live cell gate (right panels). (PDF) [file pone.0109239.s001.pdf]

Figure S2

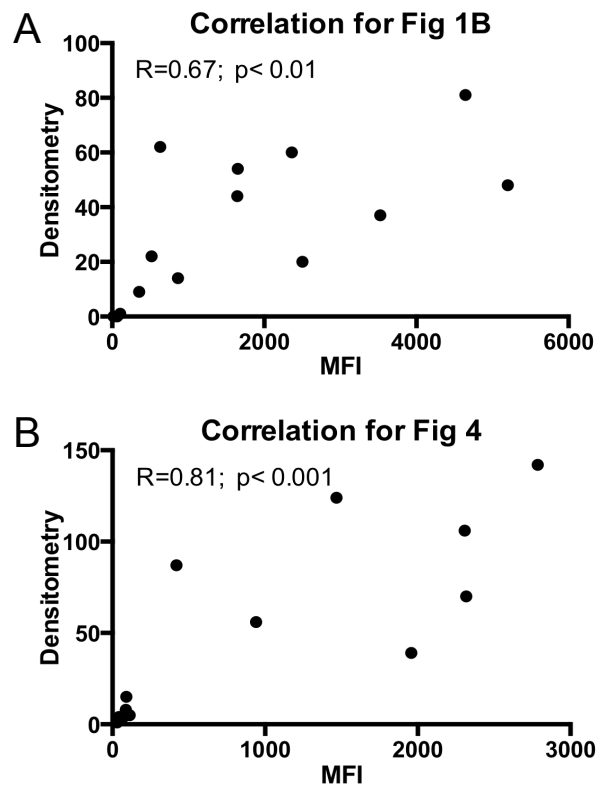

Supplement: Figure S2 — Alloantibody reactivity measured by FACS correlates with MHC-I immunoprecipitation. The intensity of immunoprecipitated MHC-I bands shown in Fig 1B and Fig 4 was analyzed by densitometry. The arbitrary grey value of the densitometry was plotted against the corresponding alloantibody binding as determined by flow-cytometry (MFI). The correlation is shown for BK cells (A) and for human lymphoblasts (B). Pearson correlation coefficients and p-values are indicated. (PDF) [file pone.0109239.s002.pdf]

Figure S3

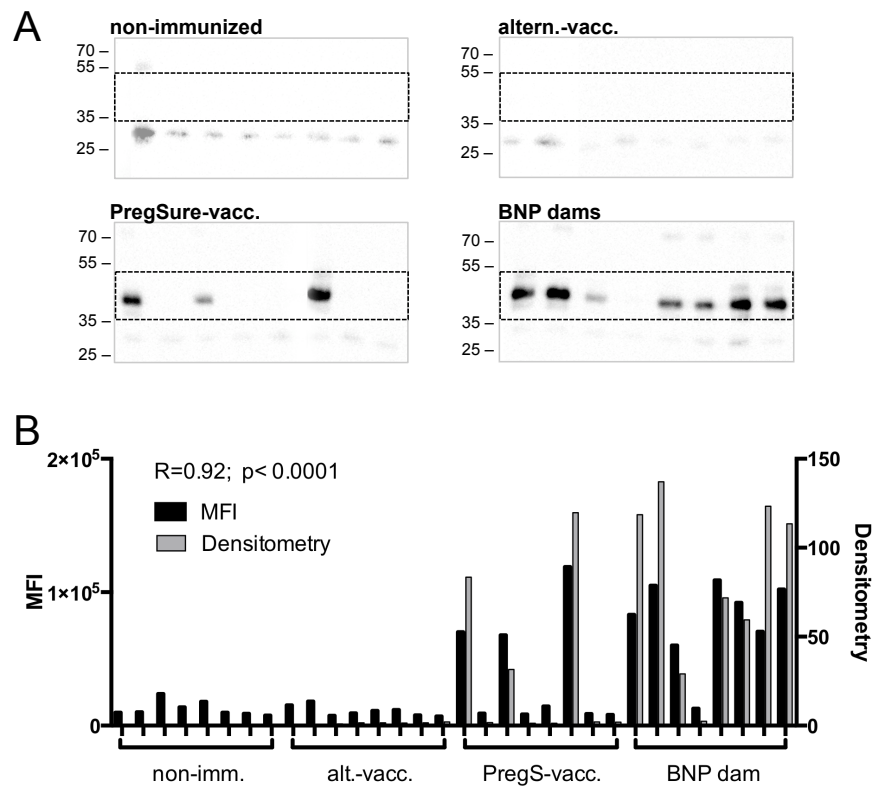

Supplement: Figure S3 — Alloantibody reactivity measured by FACS correlates with MHC-I immunoprecipitation. (A) BK cells were incubated with eight sera of non-immunized or alternatively BVD-vaccinated cows or of PregSureBVD-vaccinated non-BNP dams and PregSureBVD vaccinated BNP dams. Surface molecules were immunoprecipitated as described. The precipitates were analyzed by SDS-PAGE followed by anti BoLA-I westernblot using monoclonal antibody IL A88. Specific MHC-I bands at about 40 kDa (lined area) were analyzed by densitometry. (B) The same panel of sera was tested in parallel by flow-cytometry for alloantibody binding. MFI values (black bars) are plotted side by side with the corresponding grey values as determined by densitometry (grey bars). The correlation was calculated, the respective Pearson coefficient and the p-value are indicated. (PDF) [file pone.0109239.s003.pdf]

Figure S4

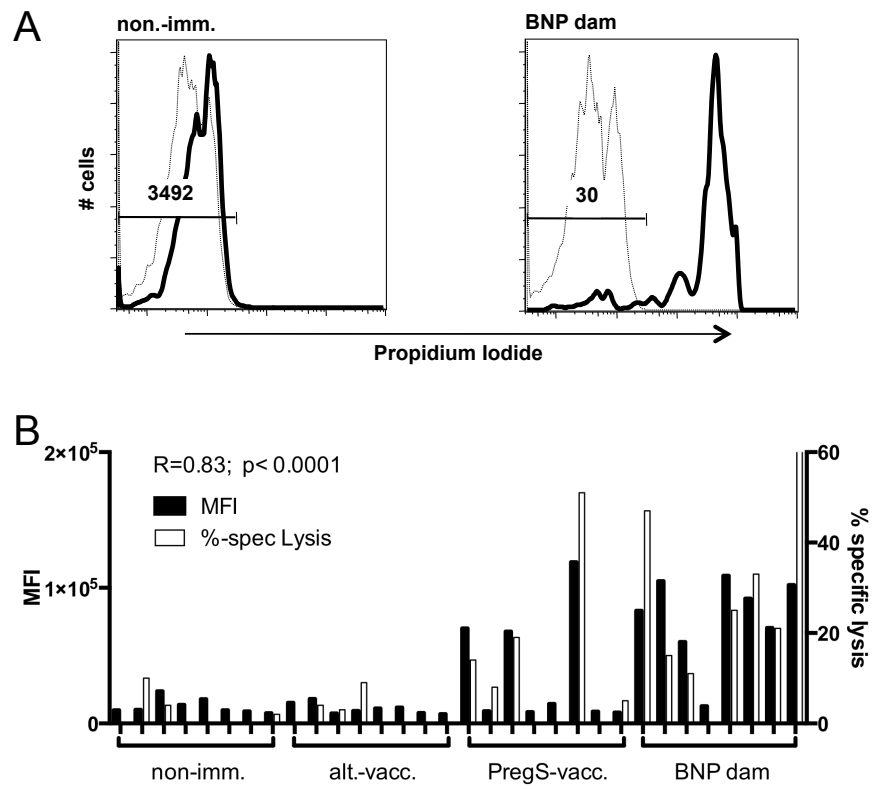

Supplement: Figure S4 — Alloantibody mediated complement lysis correlates with FACS reactivity. (A) BK cells were incubated with serum from a non-immunized cow (left panel) or a BNP dam (right panel). Heat-inactivated (dotted line) or active rabbit complement (bold line) was added and samples were incubated at 37°C. To identify dead cells red fluorescent propidium iodide was added and samples were analyzed by FACS. The absolute number of living, propidium iodide negative cells in a defined sample volume of 20 µl was determined. Numerical figures in the graphs represent the respective number of living cells after adding active complement. (B) Specific cell lysis was determined for the same serum panel as in Fig S3 and tested in parallel by flow-cytometry for alloantibody binding. MFI values (black bars) are plotted side by side with the corresponding %-specific cell lysis (open bars). The correlation was calculated, the respective Pearson coefficient and the p-value are indicated. (PDF) [file pone.0109239.s004.pdf]
